# Validating the Children’s Depression Inventory in the context of Rwanda

**DOI:** 10.1186/s12887-016-0565-2

**Published:** 2016-02-22

**Authors:** Agnes Binagwaho, Mary C. Smith Fawzi, Mawuena Agbonyitor, Sabin Nsanzimana, Corine Karema, Eric Remera, Vincent Mutabazi, Cyprien Shyirambere, Patrick Cyamatare, Cameron Nutt, Claire Wagner, Jeanine Condo, Nancy Misago, Yvonne Kayiteshonga

**Affiliations:** Ministry of Health of Rwanda, P.O. Box 84, Kigali, Rwanda; Department of Global Health and Social Medicine, Harvard Medical School, 641 Huntington Avenue, Boston, MA 02115 USA; University of Global Health Equity, 260 Blvd de l’Umuganda, P.O. Box 6955, Kigali, Rwanda; Geisel School of Medicine at Dartmouth, 1 Rope Ferry Road, Hanover, NH 03755 USA; University of Maryland School of Medicine, 685 W Baltimore St., Baltimore, MD 21201 USA; Rwanda Biomedical Center, P.O. Box 83, Kigali, Rwanda; University Teaching Hospital of Butare, Butare, Rwanda; Partners In Health, 641 Huntington Avenue, Boston, MA 02115 USA; Dana-Farber Cancer Institute Center for Global Cancer Medicine, 450 Brookline Avenue, Boston, MA 02115 USA; School of Public Health, University of Rwanda, P.O. Box 5229, Kigali, Rwanda

**Keywords:** Rwanda, Children, Adolescents, Depression, Screening, HIV, Chronic disease, Validation

## Abstract

**Background:**

Depression is often co-morbid with chronic conditions, and when combined with HIV it can increase progression and reduce survival. A brief and accurate screening tool for depression among children living with HIV is necessary to increase access to mental health care and improve HIV-related outcomes in the long-term.

**Methods:**

A validation study was conducted, comparing the Children’s Depression Inventory (CDI) with a structured clinical assessment as the gold standard among children living with HIV ages 7-14 years in Rwanda. The response rate was 87 % and the analysis was performed among 100 study participants.

**Results:**

Twenty-five percent of children had a diagnosis of depression based on the clinical interview. Sensitivity of the CDI ranged from 44 to 76 % and specificity was 92 to 100 % for cut-off scores from 5 to 9. The area under the curve (AUC) for receiver operating characteristic analysis, an estimate of overall accuracy, was 0.87 (95 % confidence interval: 0.77 – 0.97).

**Conclusions:**

The significant prevalence of depression among children living with HIV in Rwanda reflects a critical need to advance mental health care in this population. Although overall accuracy of the CDI is reasonable in this context, further research needs to be done to develop a more sensitive measure of depression in this vulnerable population. Development of a highly sensitive screening measure will be a fundamental step towards improving access to mental health care among children living with HIV, potentially improving health outcomes and quality of life in the long-term as this vulnerable population transitions into adulthood.

## Background

Depression has been shown to have a significant burden on people living with HIV (PLH) and can result in an increased risk of opportunistic infection and mortality [1, 2]. In Rwanda, antiretroviral medications (ARVs) are available throughout the country and increased survival has resulted in HIV becoming a chronic illness in this context. However, with chronic conditions there is an increased risk of depression [3, 4]. With respect to HIV, depression has been associated with reduced adherence to ARVs [5, 6] and poor quality of life [7–9]. Among children living with HIV, depression has also been documented as being co-morbid [10, 11], similar to other chronic diseases that require life-long treatment. While prioritizing the mental health needs of children living with HIV is vital to national health programs, thus far, the mental health care in this high risk population has been neglected, particularly in settings or populations with limited resources [12].

In Rwanda, the mental health needs of children are likely to be greater given the country’s history of trauma and economic insecurity [13, 14]. It has been shown that when a community experiences widespread trauma which greatly affects the social and cultural fabric, children of the following generations often continue to display symptoms of psychological trauma [15]. Depression among children has been documented in Rwanda [16] and mental health care should be a high priority, in part due to the economic hardships and the legacy of genocide that has placed their parents or caregivers at high risk of depression [17]. The history of trauma may also exacerbate the impact of HIV on children in Rwanda, where there is an estimated 22,000 children living with HIV under 15 years of age [18]. HIV-related stigma has also been observed to significantly increase the risk of depressive symptoms among youth in this context. [14] For these reasons, it is critical to identify children suffering from depression to increase access to treatment and quality of care in this vulnerable population.

Despite this critical need for care, there is currently no screening instrument for depression that has been validated among children living with HIV in Rwanda. Given the comorbidity of depression and HIV, an integrated treatment approach may increase access to mental health care and improve HIV-related outcomes [19]. An initial step towards linking mental health and HIV care among children would be the availability of a valid screening tool for depression to identify those with elevated symptoms that would benefit from treatment. This would advance access to care for depression in a system that has demonstrated increasing capacity to offer decentralized services through a network of district hospitals and community health centers. In addition, care for depression among children living with HIV would also be available through Rwanda’s progressive national health system that provides subsidized insurance for many below the poverty level. In light of the burden of depression in this at risk population and the potential for increasing access to services, the primary aim of this study is to examine the validity of a commonly used measure of depression, the Children’s Depression Inventory (CDI), among a group of children suffering from HIV receiving antiretroviral treatment in Rwanda.

## Methods

### Study design and sampling

A validation study was implemented in Rwanda from December 2011 to April 2012, comparing the CDI with depression as determined by professional psychologists trained in the use of a structured instrument based on the criteria for major depressive disorder in the DSM-IV [20] and ICD-10 [21]. Study participants were Rwandan children living with HIV, 7-14 years of age, who were aware of their HIV status and receiving antiretroviral treatment (ART) for at least 6 months.

All study participants were previously enrolled in a larger study of children living with HIV attending school that examined the effect of schooling and social support groups on treatment adherence [22]. For this broader study, a stratified random sample of 150 children living with HIV was drawn from ten health care facilities, two within each of the five Rwandan provinces. A list of eligible children was created for each of the ten facilities and numbers were assigned to each child on the list. A random number generator was then used in Microsoft Excel to randomly select 15 children from each facility. For the smaller depression validation study we randomly selected one hundred children from this larger group of 150 also using a random number generator. The response rate for completing the study questionnaires was 87 %. Given the small sample size, 13 additional children were randomly selected among the remaining 50 enrolled in the larger study to achieve the targeted sample size of 100 for the validation study.

### Measures and assessment

The Short-Form of the Children’s Depression Inventory (CDI) was selected as the tool to be validated in Rwanda given its simplicity and brevity of administration. The Short-Form of the CDI is a ten-item screening instrument with scores ranging from 0 – 20 that can be used by health workers at all levels of training and in clinical as well as non-clinical settings to identify children with elevated depressive symptoms comparable with major depression [23]. The CDI has demonstrated validity and/or reliability in a range of settings including Spain, Greece, and Germany, among other countries, with the cut-off scores varying for different countries [24]. The CDI was considered over other measures of depressive symptoms given its widespread use; it has been the most commonly used instrument to assess depression in trials among children since 1984 [24]. For the current study, The CDI was translated by a team of bilingual (English and Kinyarwanda) clinical psychologists experienced in pediatric care. The questionnaire was back-translated by a similarly qualified (but different) team. Any discrepancies were resolved at a joint meeting of these two groups, chaired by the first author of the manuscript (Dr. Agnes Binagwaho). For children, the questionnaire was piloted by clinical psychologists to ensure comprehensibility and appropriate improvements were made.

A structured clinical assessment was used as the gold standard to evaluate the validity of the CDI and identify appropriate cut-off scores for children in Rwanda. The instrument was a checklist based on the criteria for major depression in the DSM-IV [20] and the ICD-10 [21]. The clinical assessments were performed after the CDI by experienced psychologists from the National Psychosocial Center who had prior experience in pediatric mental health. If the child was diagnosed with major depression, follow-up treatment was provided by a clinical psychologist or psychiatrist at the referral hospital if needed. Informed consent was obtained through written informed consent with a parent or guardian of the child. In addition, child assent was obtained verbally. The Ethics Committee of the National University of Rwanda, which is recognized by the Rwandan National Ethics Committee, provided ethical approval.

### Statistical analysis

Descriptive statistics were calculated for sociodemographic characteristics of study participants, including frequencies for urban/rural residence, province, sex, age, education, and orphan status. Univariate analyses were conducted examining the associations between these factors and major depression. Sensitivity, specificity, positive predictive value, and negative predictive value estimates were calculated at varying cut-off scores for the CDI, using the structured clinical interview by the psychologists as the gold standard. Cut-off scores for the analysis ranged from 5 to 9, given that a specificity less than 0.90 would not offer much utility in this context. Receiver Operating Characteristic (ROC) analysis was performed to evaluate the overall validity of the CDI as compared to clinical assessment in the Rwandan context.

## Results and Discussion

### Results

Table 1 presents the sociodemographic characteristics of study participants. Fifty-seven percent of the participants were female and 93 % were currently enrolled in primary school. Thirty-three percent of the participants were from urban settings and 20 % were from each of the five provinces, in correspondence with the sampling strategy for the study. Table 2 summarizes the distribution of sociodemographic characteristics by children who were depressed versus not depressed according to the structured clinical interview. Based on this assessment, 25 % of the children had a clinical diagnosis of depression. A statistically significant finding was observed by province, whereby 75 % of the children in the Western Province were depressed, as compared to 5 % in Kigali City (*p* < 0.001). Other potentially meaningful differences were observed between groups, although they did not achieve statistical significance. For example, a trend was observed whereby over 28 % of participants residing in rural areas were depressed versus 18 % in urban areas (*p* = 0.331). Nearly 30 % of girls were depressed versus 19 % for boys, although this finding was not statistically significant (*p* = 0.247).

**Table 1:** Sociodemographic characteristics of children living with HIV

Characteristic	% (*n* = 100)
**Age**	
7-9	23
10-12	39
13-14	38
**Sex**	
Male	43
Female	57
**Education level**	
Primary	93
Secondary	6
Vocational	1
**Residence**	
Urban	33
Rural	67
**Province**	
East	20
Kigali City	20
North	20
South	20
West	20
**Orphan status**	
No	35
Yes – single orphan	46
Yes – double orphan	19
**Depression**	
Depressed	25
Not depressed	75

**Table 2:** Characteristics of respondents by depression according to clinical assessment (*n* = 100)

Characteristic	Depressed *n* = 25		Not Depressed *n* = 75		p-value* (degrees of freedom)
	n	%	n	%	
**Sex**					0.247 (1)
Male	8	18.6	35	81.4	
Female	17	29.8	40	70.2	
**Education level**					0.108 (2)
Primary	24	25.8	69	74.2	
Secondary	0	0	6	100	
Vocational	1	100	0	0	
**Residence**					0.331 (1)
Urban	6	18.2	27	81.8	
Rural	19	28.4	48	71.6	
**Province**					<0.001 (4)
East	2	10.0	18	90.0	
Kigali City	1	5.0	19	95.0	
North	2	10.0	18	90.0	
South	5	25.0	15	75.0	
West	15	75.0	5	25.0	
**Orphan status**					0.790 (2)
No	10	28.6	25	71.4	
Yes – single orphan	10	21.7	36	78.3	
Yes – double orphan	5	26.3	14	73.7	

*Due to the small sample size Fisher’s exact test was used

**Fig. 1:**
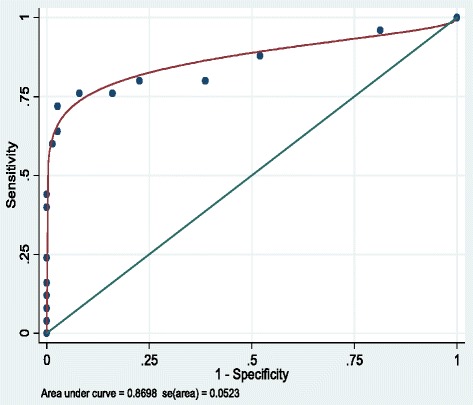
ROC analysis – Children’s Depression Inventory

Table 3 provides estimates of sensitivity, specificity, positive predictive value, and negative predictive value at varying cut-off scores of the CDI using the clinical assessment as the gold standard. The sensitivity ranged from 76 to 44 % for cut-off scores of 5 and 9, respectively. For specificity, estimates were from 92 to 100 % for the same cut-off scores. Based on the ROC analysis, the area under the curve (AUC) was 0.87 (95 % CI: 0.77 – 0.97), demonstrating a fair degree of accuracy of the CDI as compared to a structured diagnostic interview as the gold standard (see Fig. 1).

**Table 3:** Sensitivity, specificity, positive predictive value, and negative predictive value of depression (95 % CIs) for various CDI cut-off scores

			CDI Cutoff							
	>5	≤5	>6	≤6	>7	≤7	>8	≤8	>9	≤9
**Depression**										
**Yes**	19	6	18	7	16	9	15	10	11	14
**No**	6	69	2	73	2	73	1	74	0	75
**Sensitivity**	0.76 (0.57, 0.89)		0.72 (0.52, 0.86)		0.64 (0.45, 0.80)		0.60 (0.41, 0.77)		0.44 (0.25, 0.63)	
**Specificity**	0.92 (0.84, 0.96)		0.97 (0.91, 0.99)		0.97 (0.91, 0.99)		0.99 (0.93, 1.0)		1.0 (0.95, 1.0)	
**Positive predictive value**	0.76 (0.57, 0.89)		0.90 (0.70, 0.97)		0.89 (0.67, 0.97)		0.94 (0.72, 0.99)		1.0 (0.74, 1.0)	
**Negative predictive value**	0.92 (0.84, 0.96)		0.91 (0.83, 0.96)		0.89 (0.80, 0.94)		0.88 (0.80, 0.93)		0.84 (0.75, 0.90)

### Discussion

Children living with HIV are at risk of major depression [10, 11], due in part to the social isolation and stigma that often accompanies the disease as well as remaining uncertainty about their future. In Rwanda, since ART is more widely available for children living with HIV, the disease has become a chronic condition. Depression is often co-morbid with chronic illness [3, 4], and as HIV-related survival has improved substantially it is important to consider strategies for identifying and treating major depression among children living with HIV. Our findings demonstrate the feasibility of administering a screening measure for depressive symptoms among children receiving HIV treatment in Rwanda. Based on ROC analysis, the overall accuracy of the CDI was good (AUC = 0.87). A cut-off score of 5 demonstrated reasonable estimates of sensitivity (76 %) and specificity (92 %).

Although the CDI has been used in several studies in sub-Saharan Africa [25, 26], it has not yet been validated in Rwanda. Given that the presentation of symptoms and interpretation of severity may vary across cultures, it is important to validate measures of psychological symptoms in different contexts, to ensure that the measure is identifying youth with elevated symptoms comparable with major depression with a reasonable degree of accuracy [27]. For example, studies of the CDI among adolescents from China [28] and Puerto Rico [27] reflected the need for different cut-off scores for a level of symptoms comparable with major depression in different cultural contexts.

Findings from this study indicated that sociodemographic factors such as urban/rural residence, sex, and educational level were not associated with depression in this population. This may be due to limited sample size, given that expected trends were observed for some variables [29, 30]. However, children receiving ART in the Western Province were at the highest risk of depression. Consideration of geographical variation in the burden of depression in this population may be necessary in planning the roll out of services that integrates depression screening activities within the context of HIV care for children. Research is currently underway to better understand why the level of depression is higher in some of the geographic areas, examining factors such as history (e.g., impact of genocide) and other health risks (e.g., malnutrition). Poverty may also play a role, since children receiving ART in rural areas were at higher risk of depression (28 %) as compared with those in urban settings (18 %), although this finding was not statistically significant. Broader initiatives in poverty reduction in Rwanda, a key priority for the government through its Vision 2020 plan, may in the long-term have an effect on the burden of depression among children living with HIV.

The CDI demonstrated a higher specificity in this population (92 %) compared to the sensitivity (76 %). This is to ensure that false-positives are minimized given limited resources for mental health care in Rwanda. However, the lower sensitivity indicates that over 20 % of true-positives will also be missed, which should be a consideration for future studies. Inclusion of locally derived depressive symptoms may increase the sensitivity of a screening tool. Although a recent validation study of the Center for Epidemiologic Studies Depression Scale for Children (CES-DC) that included symptoms developed locally was conducted with youth in Rwanda ages 10-17, this was performed among the general population as opposed to children living with HIV [16].

There are some important limitations to this study. First, given its small sample size, it is not possible to determine if the null findings noted above are due to a true lack of association or to limited statistical power. A greater number of study participants would improve our power to detect an association between depression and sociodemographic characteristics of interest. However, since this study relied upon in-depth clinical evaluations, it would not have been feasible to recruit a large number of children in our study population. In addition, due to the sample selection process among children living with HIV receiving treatment, there was not an equal number of depressed versus non-depressed participants. This also limits the statistical power of our study. In addition, since this validation study was conducted among children living with HIV who also are actively receiving care, the findings may not be generalizable to children who may not be accessing treatment. Finally, locally relevant symptoms of depression were not included in the symptom scale. This may have potentially reduced the sensitivity of identifying cases of major depression.

A screening tool with higher sensitivity would offer greater utility among children living with HIV, given the significant burden of depression in this population (25 % prevalence). There is a need to develop Rwandan policy to address routine screening for depression among children living with HIV. Improving mental health services for this segment of the population may result in improved adherence and survival [1, 5]. Moreover, such actions are likely to improve quality of life [7] and children’s hope for the future.

The potential for increased health benefits of treating depression for children with other chronic conditions may also be evident given the burden in those populations [3, 31–33]. Therefore, it is important to screen for depression in children suffering from all chronic diseases, not only HIV. By using a quick, easy-to-interpret depression screening tool, this goal is achievable in Rwandan national health policy. Moreover, such a goal is revolutionary, as no African government has yet dedicated resources to routine screening of children at risk for depression, nor has a policy been put in place that will systematically provide treatment to those in need. This study is an important step in realizing such an objective.

## Conclusions

This study demonstrated that depression is often co-morbid with HIV among children in Rwanda, indicating the need for integrating mental health care with HIV treatment for youth in this context. In order to identify children living with HIV at risk for depression, a brief and accurate screening measure is needed for use at the point at which care is delivered. Although the CDI reflected an overall good degree of accuracy as well as reasonable sensitivity and specificity, increasing the sensitivity of the measure by adding locally derived symptoms may help improve this screening instrument for depression among children living with HIV in Rwanda. Access to a highly sensitive screening measure for depression will be a fundamental step towards improving access to mental health care among children living with HIV, potentially improving HIV-related outcomes and quality of life in the long-term as this vulnerable population transitions into adulthood.

## Abbreviations

ART: antiretroviral therapy
ARVs: antiretroviral medications
AUC: area under the ROC curve
CDI: Children’s Depression Inventory
DSM-IV: Diagnostic and Statistical Manual for Mental Disorders, version IV
ICD-10: International Statistical Classification of Diseases, version 10
PLH: people living with HIV
ROC: receiver operating characteristic
